# Electroanatomical mapping-guided leadless pacemaker (MICRA-VR) implantation in the right atrium of a univentricular heart with Fontan circulation, an approach combining imaging and electrophysiology: insights from a case report

**DOI:** 10.1093/ehjcr/ytaf568

**Published:** 2025-11-10

**Authors:** Clémence Chevalier, Océane Rea, Nawel Babouri, Francis Bessière, Christelle Haddad

**Affiliations:** Service de Rythmologie Cardiaque, Hôpital Cardiologique Louis Pradel, Institut de Cardiologie des Hospices Civils de Lyon, 28 avenue du Doyen Lépine, Bron 69500, France; Service de Rythmologie Cardiaque, Hôpital Cardiologique Louis Pradel, Institut de Cardiologie des Hospices Civils de Lyon, 28 avenue du Doyen Lépine, Bron 69500, France; Service de Rythmologie Cardiaque, Hôpital Cardiologique Louis Pradel, Institut de Cardiologie des Hospices Civils de Lyon, 28 avenue du Doyen Lépine, Bron 69500, France; Service de Rythmologie Cardiaque, Hôpital Cardiologique Louis Pradel, Institut de Cardiologie des Hospices Civils de Lyon, 28 avenue du Doyen Lépine, Bron 69500, France; Université Claude Bernard Lyon 1, 8 avenue Rockfeller, Lyon 69003, France; Laboratoire des applications thérapeutiques des ultrasons, LabTAU INSERM U1032, 152 Cours Albert Thomas, Lyon 69003, France; Service de Rythmologie Cardiaque, Hôpital Cardiologique Louis Pradel, Institut de Cardiologie des Hospices Civils de Lyon, 28 avenue du Doyen Lépine, Bron 69500, France

**Keywords:** Atrium, Leadless pacemaker, Tricuspid atresia, Case report

## Abstract

**Background:**

Patients with complex congenital heart disease, especially after Fontan repairs, face significant challenges in managing conduction disorders. This case illustrates the potential of leadless pacemaker technology, which is particularly relevant and promising in these patients, by combining an unusual implantation site with electroanatomical guidance.

**Case summary:**

This report describes a 41-year-old male with complex congenital heart disease, including tricuspid atresia repaired using a historical Fontan circulation (with the right atrial appendage connected to the pulmonary trunk). Severe right atrial dilation led to drug-refractory arrhythmias that necessitated several catheter-based ablation procedures. Additionally, the patient developed sinus node dysfunction and ultimately required pacemaker implantation. Due to anatomical challenges and extensive scarring in the right atrium, a leadless pacemaker (MICRA-VR) was implanted using both fluoroscopic and three-dimensional electroanatomical mapping techniques. At follow-up, the patient remained asymptomatic, with stable device positioning.

**Discussion:**

This off-label technique, which helps prevent device-related infections and venous system occlusion, should be considered a viable option in complex cases as leadless pacemaker technology continues to advance.

Learning points

- Leadless pacemakers represent an effective alternative to transvenous or epicardial systems in congenital heart disease patients, overcoming most long-term-related complications.
- Implantation of tine-based leadless pacemakers in the atrial position is an off-label procedure but could be considered in carefully selected patients.
- Electroanatomical mapping enables precise placement by identifying viable myocardial tissue, which is critical in anatomically complex or surgically modified hearts.

## Introduction

Patients with complex congenital heart disease, particularly those with Fontan repairs, present unique challenges for the management of conduction disorders. Emerging leadless pacemaker technologies provide new strategies to navigate complex anatomies while reducing the long-term complications associated with traditional epicardial or transvenous systems.

## Summary figure

**Figure:**
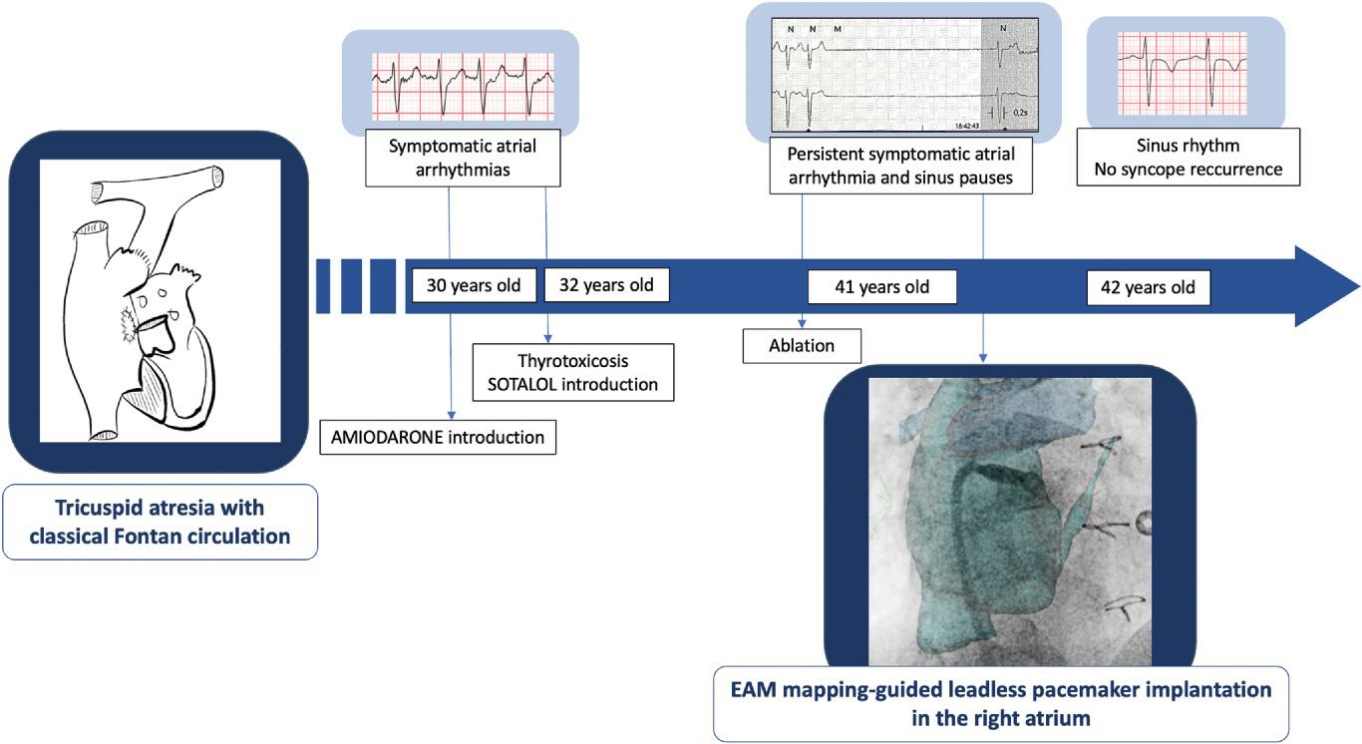


## Case presentation

**Figure 1:**
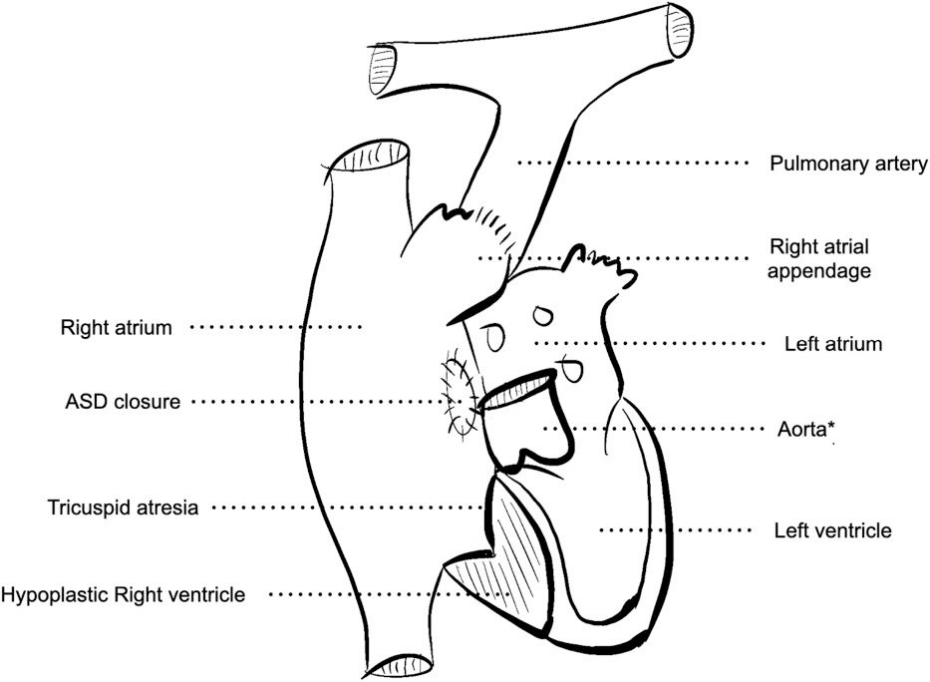
Schematic representation of tricuspid atresia after the historical Fontan procedure with right atrial appendage-to-pulmonary artery anastomosis, atrial septal defect closure and a hypoplastic right ventricle. ASD; atrial septal defect. *For schematic purposes, the aorta has been cut just after the sinotubular junction to prevent overcrowding of the diagram.

This report describes a 41-year-old male with complex congenital heart disease, including tricuspid atresia and atrial septal defect, resulting in a markedly hypoplastic right ventricle. He underwent surgical classical Fontan procedure, with anastomosis of the right atrial appendage to the pulmonary artery and closure of the atrial septal defect. Systemic venous return was thus directed into the right atrium and subsequently drained directly into the pulmonary artery (*Figure 1*).

**Figure 2:**
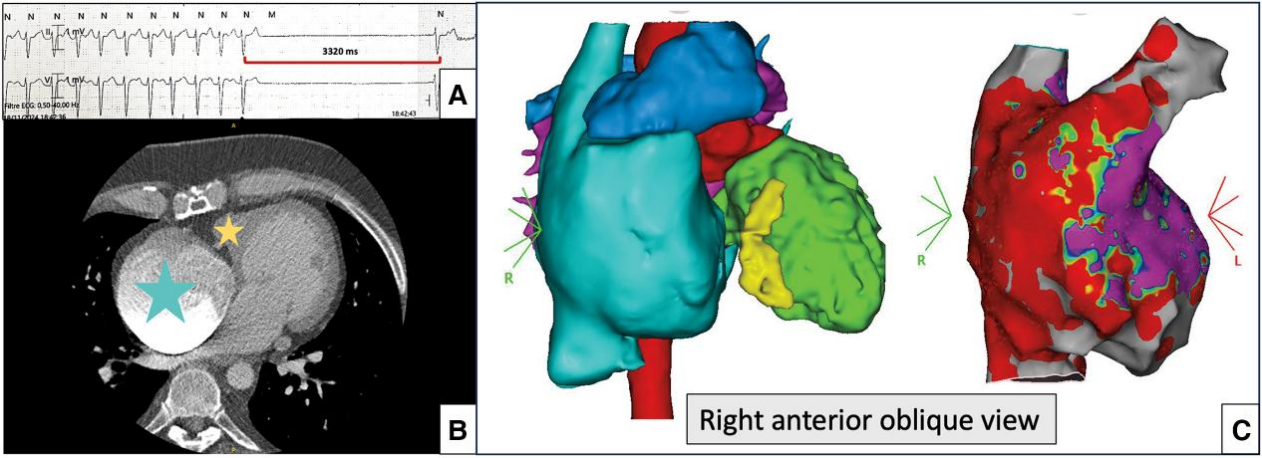
(*A*) Electrocardiogram (25 mm/s) showing sinus node dysfunction. (*B*)Axial computed tomography scan demonstrating severe right atrial dilatation (big/blue star) and a right ventricular hypoplasia (little/yellow star) resulting from tricuspid atresia. (*C*) Three-dimensional reconstruction of the heart from computed tomography scan (left): right atrium (the largest cavity, light blue), pulmonary artery (on top, blue), left ventricle (right, green), right ventricle (yellow, between the right atrium and the left ventricle), and aorta (back, red). Voltage electroanatomical mapping (right) of the right atrium in sinus rhythm showing low-voltage zones suggestive of extensive scar tissue. CT, computed tomography; 3D, three-dimensional.

The patient subsequently developed severe right atrial dilation, which led to recurrent, symptomatic atrial arrhythmias in his 30s. Management was complicated by amiodarone-induced thyrotoxicity that required thyroidectomy 2 years later. For almost 10 years, rhythm control was achieved with sotalol (160 mg/day), until atrial flutter recurred with sinus node dysfunction. Despite ablation, antiarrhythmic therapy remained necessary, further exacerbating sinus pauses (up to 8 s), occasionally associated with syncope (*Figure 2*). This prompted the indication for pacemaker implantation.^1^ An ‘ablate-and-pace’ strategy was discussed as an alternative therapeutic option; however, ventricular pacing was challenging and posed a risk of inducing pacing-related ventricular dysfunction. Ultimately, an isolated atrial pacemaker was favoured, as the patient had no atrio-ventricular (AV) conduction disorder, allowing for the continued use of antiarrhythmic therapy. Given the patient’s young age and the need to minimize lead-related complications, a leadless pacemaker was preferred. At the time of treatment, a dedicated atrial leadless device was not available; therefore, after discussing options with the patient, the MICRA-VR system was selected.

**Figure 3:**
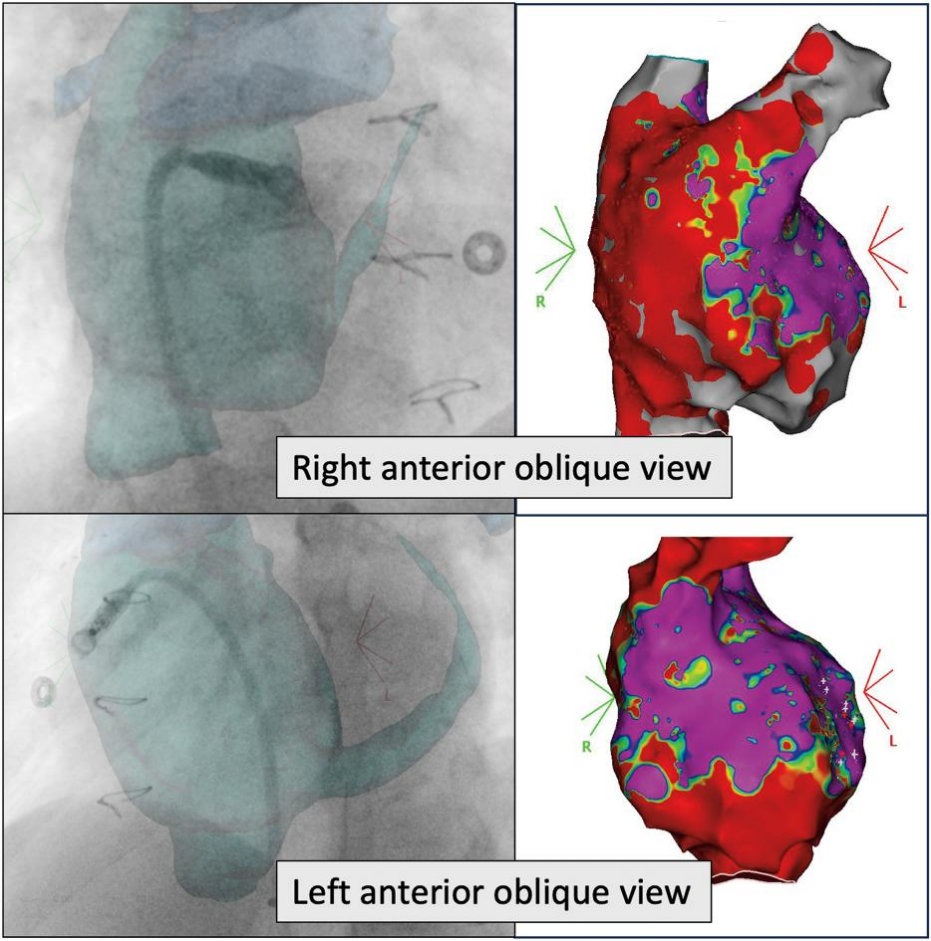
Fluoroscopic (left) and electroanatomical voltage map (right) images of the right atrium showing implantation of the leadless pacemaker in the right appendage, in the right anterior oblique and left anterior oblique views. RAO, right anterior oblique; LAO, left anterior oblique.

The procedure was performed under local anaesthesia using right femoral venous access. Initially, staged angiography through the delivery sheath was used to visualize the trabeculated atrial appendage and the anatomy of the pulmonary artery anastomosis. In addition to fluoroscopic guidance, electroanatomical mapping (EAM) identified healthy tissue within a large fibrotic area, ensuring optimal pacemaker placement. Once the device was correctly positioned and the delivery system released, the pacemaker was successfully implanted in the atrial appendage on the first attempt (*Figure 3*). The final parameters were: an A detection amplitude of 2 mV, an impedance of 790 Ω, and an A pacing threshold of 1.75 V at 0.24 ms. Additional fluoroscopic views confirmed the device’s stability with no signs of dislodgement, and echocardiography revealed no pericardial effusion. At follow-up, the patient remains asymptomatic on sotalol, with atrial stimulation occurring only 1% of the time with a minimal frequence at 50 b.p.m. during the 6-month review.

## Discussion

Leadless pacemakers have been developed as an alternative to traditional transvenous pacemakers to overcome pocket or lead-related complications and venous access issues. These considerations are of particular importance in patients with congenital heart diseases. Moreover, these devices are expected to offer greater longevity, and these patients are usually young.^2^

An atrial device was preferred in this patient due to his anatomy (right ventricle hypoplasia) and preserved atrioventricular conduction. Atrial leadless pacemakers have recently been developed with an activation fixation delivery system. However, these devices are currently available in only a few centres worldwide.^3^

The tine-based fixation is designed to anchor in trabeculated right ventricles. A recent pre-clinical study tested the implantation of those devices in four minipigs.^4^ Pacing performance and stability were satisfactory and remained stable at 3 months. However, open-heart evaluations revealed notable myocardial penetration by the device tines, though without pericardial effusion or inflammation. This risk may be lower in patients with congenital heart disease who have undergone surgery, as long-term cardiac barometric stress can lead to myocardial thickening, including the atrial wall.^5^ Additionally, pericardial remodelling following open-pericardium surgery, as in this patient, reduces the risk of tamponade.^6^

The first cases of atrial leadless pacemaker implantation with tined fixation were reported in two patients with Mustard atrial switch repair.^7^ An anatomic approach was used, with iodine injection guidance through the left femoral vein to place the device in the left atrial appendage. A similar case was described in a patient with Ebstein’s disease and sinus node dysfunction, in whom right atrial appendage implantation was performed after failed ventricle placement.^8^ In both reports, electrical efficacy was confirmed without pericardial complications at 6 and 12 weeks, respectively. The highly dilated atria in these patients facilitated manipulation of the delivery system and the significantly trabeculated appendage, fixation, and stability of the device.

Electroanatomical mapping has been rarely reported as a tool for pacemaker implantation to limit X-ray exposure^9^ and/or to facilitate device placement in complex anatomy. Its use has been described for transvenous leads after Fontan palliation and for conduction system pacing in transposition of the great arteries with dextrocardia.^10^ In our case, direct connection of the leadless device to the mapping system was not feasible. Nevertheless, EAM facilitated navigation within the enlarged atrium and enabled identification of limited areas of healthy myocardium suitable for pacing. Despite additional cost and time, EAM provides distinct advantages in complex anatomy by improving anatomical understanding, avoiding scarred tissue, and ensuring stable and effective device positioning.

By the end of device lifespan, the patient will be ∼55 years old.^11^ Leadless atrial pacemaker extraction and re-implantation would be preferred, despite limited long-term safety data.^12^ Additional implants remain feasible within the enlarged tight atrium, as up to three may coexist within the same cavity.^13^ A conventional atrial pacemaker is an alternative. If atrial arrhythmias become permanent, atrial pacing would no longer be necessary, and a ‘pace-and-ablate’ strategy with conduction system pacing would be favoured.^14^ Electroanatomical mapping would be valuable in this scenario^10^ to localize the conduction system from the atrioventricular node to the left fascicles, given the anatomical variations described in tricuspid atresia.^15^

This innovative approach opens the door to considering the use of the MICRA-VR in the atrial position. However, it must be evaluated on a case-by-case basis, as this technique remains off-label. Leadless pacemakers with tine-based fixation are currently approved only for right ventricle use. Employing these devices for an unintended purpose introduces potential risks that must be carefully evaluated.

This case underscores the necessity of evolving technologies. It emphasizes the place of EAM system to enhance safety and precision of pacemaker implantation in complex heart disease. The use of leadless atrial pacemakers will soon be eased with the development of dedicated devices, such as atrial or dual-chamber leadless pacemakers capable of communicating via wireless technology (AVEIR, Abbott). However, challenges remain for congenital heart disease patients, as these systems require relatively straight venous access path, and their long-term extractability remains unresolved.

## Data Availability

Data available on request—the data underlying this article will be shared on reasonable request to the corresponding author.

## Lead author biography

Clémence Chevalier is a 26-year-old cardiology resident. She is in her third year of training at the University Hospital of Lyon (France). Her areas of interest include heart failure, cardiomyopathies, and innovations.
